# Fuzzy Performance between Surface Fitting and Energy Distribution in Turbulence Runner

**DOI:** 10.1100/2012/408949

**Published:** 2012-11-04

**Authors:** Zhongwei Liang, Xiaochu Liu, Bangyan Ye, Richard Kars Brauwer

**Affiliations:** ^1^School of Mechanical and Electrical Engineering, Guangzhou University, Guangzhou 510006, China; ^2^School of Mechanical and Automotive Engineering, South China University of Technology, Guangzhou 510640, China; ^3^Department of Information Engineering, Indian Institute of Technology (IIT), Kanpur 208023, India

## Abstract

Because the application of surface fitting algorithms exerts a considerable fuzzy influence on the mathematical features of kinetic energy distribution, their relation mechanism in different external conditional parameters must be quantitatively analyzed. Through determining the kinetic energy value of each selected representative position coordinate point by calculating kinetic energy parameters, several typical algorithms of complicated surface fitting are applied for constructing microkinetic energy distribution surface models in the objective turbulence runner with those obtained kinetic energy values. On the base of calculating the newly proposed mathematical features, we construct fuzzy evaluation data sequence and present a new three-dimensional fuzzy quantitative evaluation method; then the value change tendencies of kinetic energy distribution surface features can be clearly quantified, and the fuzzy performance mechanism discipline between the performance results of surface fitting algorithms, the spatial features of turbulence kinetic energy distribution surface, and their respective environmental parameter conditions can be quantitatively analyzed in detail, which results in the acquirement of final conclusions concerning the inherent turbulence kinetic energy distribution performance mechanism and its mathematical relation. A further turbulence energy quantitative study can be ensured.

## 1. Introduction

Through searching the academic literature published in recent years we learn that resulting from the rapid progress of turbulence science, the complexity and spatial meaning of objective fluid characteristic have already broken the traditional sense, and the precise turbulence monitoring has touched upon energy distribution domain in its flow runner; furthermore, we found that the optimization design and arrange deployment on reversing valve runner wall play an important influence effect on the following model construction of kinetic energy distribution in practice. Although surface fitting provides crucial theoretical foundations for precision machining, the performance assessment between surface fitting algorithms and turbulence kinetic energy distribution in different parameter conditions still remains unstudied and needs further detailed investigations.

In the research area of microturbulence energy distribution, some original papers have been published in the topic of energy spectra measurement on a given complicated turbulence fluid section during the past several years, which provide a new investigation idea for turbulence fluid structure modeling [1–3]. For example, there are some researchers who focus on the comparison of the liquid energy spectra and velocity probability density functions with experimental data obtained by phase-sensitive constant-temperature anemometry [4]. Simultaneously, Liu et al. [5] studied the statistical properties of complex fluid field networks which were constructed from energy distribution in three-dimensional fully developed turbulence runner by using the visibility algorithm. All these works provide original ideas and science references for our further researches. In the subject of microfluid section feature analysis and kinetic energy distribution modeling, Panidis [6] has investigated the topic of turbulent flow field kinetic energy generated due to the interaction of grid turbulence pressure in a vertical channel of rectangular cross-section. More relevant theoretical progress can also be found in [7–9]. It can be seen that these traditional research results still keep a considerable distance from microfluid section kinetic energy characteristics and their respective fitting algorithms that being paid attention to, which become our research interest in this paper. With the help of three-dimensional fluid modeling, Ahmed [10] employed laser Doppler velocimeter to measure and model the three-dimensional flow properties of a confined, isothermal, swirling flow field in an axisymmetric sudden expansion research combustor. Fujiwara et al. [11] investigated the statistical characteristics of spatial distribution fluctuations of kinetic energies of each component wave and its time derivative in wave turbulence for a Hamiltonian system with a nondecay type dispersion relation. We can also learn other similar investigations and representative results from [12–14]. Most of them focused on the fluid mechanics analysis in a narrow theoretical sense which describes the studied turbulence energy distribution properties without any detailed consideration on its three-dimensional structure features, the mutual fuzzy performance mechanisms between fluid energy distribution models and their respective surface fitting algorithms, leaving these difficult problems unsolved which should be further investigated.

This paper is structured as follows.  Section 1 outlines the importance and necessity of three-dimensional fuzzy evaluation of the influence mechanism between surface fitting and turbulence kinetic energy distribution in a reversing valve runner; Section 2 explains the detailed computation process and theoretical basis of turbulence kinetic energy; Section 3 describes some newly proposed mathematical features for accurately describing energy distribution surface models.  Section 4 presents an experimental process and illustrates the computed results; then a detailed fuzzy mutual-influence analysis and quantitative data evaluation can be made in Section 5 with the newly proposed three-dimensional fuzzy evaluation method after performance comparison and suggestion presentation in the specific experimental process Section 6 concludes this paper as required.

## 2. Turbulence Kinetic Energy

Consider the fact that the inner structure of reversing valve runner is characterized by complicated topography, ultraminiature size, and high-pressure closed condition, which explains the difficulties of describing the dynamic fluid properties. A microcomputerized runner model simulating a high-pressure reversing valve was obtained by computer-aided design, and then its solid model in 1 : 1 size scale can easily be produced by rapid forming/fast molding when using transparent high-polymer PVC material for clearly demonstrating the inner microtopography of target runner space and its subsequent turbulence moving process promptly.

In this paper, we use fluid function as the most frequentlyused model to compute turbulence kinetic energy according to its inherent mechanism. In order to clearly describe the detailed computation condition and dynamic process of kinetic energy, we place microfluid sensors on the inner wall of valve runner, which contributes to the acquirement of turbulence parameter (time duration, fluid velocity, viscosity, pressure, temperature, concentration, density, and things like that vary with time and space) from different measurement positions; afterwards, we define turbulence kinetic energy *K* in the *i*th sampling time interval, by recording turbulence flow signal such as velocities, motion vectors, and flow directions in each instantaneous monitor position; it is supposed that the statistic time moments should be denoted as *T* = {*t*_1_, *t*_2_, *t*_3_,…, *t*_*n*_}. With the amount of sampling levels being denoted as *n*, then turbulence average velocity in one presumptive time interval $\bar{\mu_{i}}$ can be computed as [15–17]

(1) $$\begin{array}{c} \bar{\mu_{i}}=\frac{1}{n}\sum_{i=1}^{n}\left[\frac{1}{T}\int_{0}^{T}\mu_{i}\left(t\right)dt\right]. \end{array}$$

Here *μ*_*i*_(*t*) denotes the turbulence instantaneous velocity in the time moment of *t* which locates in the *i*th sampling time interval. Thus turbulence intensity *μ*_*i*_′ in one presumptive time interval can be computed as

(2) $$\begin{array}{c} {\mu_{i}}^{\prime }=\frac{1}{n}{\sum_{i=1}^{n}\left[\frac{1}{T}\int_{0}^{T}{\left[\mu_{i}\left(t\right)-\bar{\mu_{i}}\right]}^{2}dt\right]}^{1/2}. \end{array}$$

Here the dissipation rating *ε* is defined as

(3) $$\begin{array}{c} \varepsilon =\mu_{t}{\left[\frac{\partial \mu_{i}}{\partial x}\right]}^{2}=C_{D}\frac{K^{3/2}}{\lambda }. \end{array}$$

Turbulence kinetic energy *K* of the *i*th sampling time interval can be defined by following parametric equations:

(4) ρ∂K∂t+ρμi∂K∂xi=∂∂xi[(μ+μtσε)∂K∂xi]+μt∂μt∂xi(∂μt∂xi+∂μt∂yi+∂μt∂zi)−ρε,ρ∂K∂t+ρμi∂K∂yi=∂∂yi[(μ+μtσε)∂K∂yi]+μt∂μt∂yi(∂μt∂xi+∂μt∂yi+∂μt∂zi)−ρε,ρ∂K∂t+ρμk∂K∂zi=∂∂zi[(μ+μtσε)∂K∂zi]+μt∂μt∂zi(∂μt∂xi+∂μt∂yi+∂μt∂zi)−ρε.

Coefficient of the turbulence's viscosity *μ*_*t*_ is given as

(5) $$\begin{array}{c} \mu_{t}={C_{\mu }^{}}^{\prime }\rho K^{1/2}L=\left({C_{\mu }^{}}^{\prime }+C_{D}\right)\rho K^{2}\frac{L}{C_{D}K^{3/2}}=\frac{{C_{\mu }^{}}^{\prime }\rho K^{2}}{\varepsilon }. \end{array}$$

Here *C*_*μ*_′ is an empirical factor, *K* is the kinetic energy value to be computed, and *L* is the length scale of turbulence movement. When integrating both sides of the differential equations, turbulence kinetic energy *K* can be finally obtained as

(6) K=12(μiμt¯)=(12n∑i=1n[1T∫0T[μi(t)−μi¯]2dt]1/2)×Cμ′ρε×1nμ′2∑i=1n[1T−τ∫0T−τ[μi(t)μi(t+τ)]dt]=Cμ′ρ2εn2μ′2(∑i=1n{[1T∫0T[μi(t)−μi¯]2dt]1/2        ×[1T−τ∫0T−τ[μi(t)μi(t+τ)]dt]})=Cμ′ρ2εn2μ′2T(T−τ)×∑i=1n{[∫0T[μi(t)−μi¯]2]1/2[μi(t)μi(t+τ)]}dt.

This result can be regarded as the computed turbulence kinetic energy in reversing valve runner as the objective target [18, 19]. 

## 3. Mathematical Features of Energy Distribution Surface

As we use several typical surface fitting algorithms in this experiment, such as surface of NURBS, energy optimization modeling, B-spline of quasiuniform bicubic, trigonometry Bernstein-Bezier, and scattered data interpolation, the following mathematical features are newly proposed for describing our fitting results.

**Feature 1**

Consider

(7) φ=α1∑i=1mWui2+β1∑i=1mWuui2+α2∑j=1nWvj2+β2∑j=1nWvvj2+α1α2β1β2∑i=1m∑j=1nWuivj2−2f(u,v)W.

Here *W* is one constructed surface in the form of B-spline primary function; *W*_*u*_, *W*_*v*_, *W*_*uu*_, *W*_*vv*_, *W*_*uv*_ are the partial derivatives of the objective fitted surface *W* in the first order, second order, and hybrid state of *u*, *v* axes, respectively; *α*_1_, *α*_2_, *β*_1_, *β*_2_ are given parameters, and *f*(*u*, *v*) is a given function of surface vector, *m*, *n* are the order numbers of surface vector of *u*, *v* axes [20].

**Feature 2**

Consider

(8) ζ=∑i=1m∫ΩSu(ui)2du+∑i=1m∑i=1m∬i∈ΩSuu(uii)2du du+∑j=1n∫ΩSv(vj)2dv+∑j=1n∑j=1n∬j∈ΩSvv(vjj)2dv dv+∑i=1m∑j=1n∬ΩSuv(uivj)2du dv.

Here *S*_*u*_(*u*_*i*_), *S*_*uu*_(*u*_*ii*_), *S*_*v*_(*v*_*j*_), *S*_*vv*_(*v*_*jj*_), *S*_*uv*_(*u*_*i*_ *v*_*j*_) are the first order, second order, and hybrid derivatives of surface *f*(*u*, *v*) in *u*, *v* axes.

**Feature 3**

Consider

(9) ρ=−2∑i=0,muu∑j=0,mvvVi,j∯i,j∈ΩNi,su(u) ∗Nj,sv(v)Ni,j(uv)f(u,v)du dv.

Here *N*_*i*,*s*_*u*__(*u*), *N*_*j*,*s*_*v*__(*v*), *N*_*i*,*j*_(*uv*) are the boundary control B-spline surface in *u*, *v*, *uv* axes, respectively; *f*(*u*, *v*) is a given vector function, with *V*_*i*,*j*_ denoting the transitional vector obtained from the surface external load.

**Feature 4**

Consider

(10) $$\begin{array}{c} Z_{nm}=\frac{n+1}{\pi }\int_{0}^{1}\int_{0}^{2\pi }R_{nm}\left(\gamma \right)e^{jm\theta }f\left(\gamma ,\theta \right)\gamma \;d\gamma \;d\theta . \end{array}$$

Here *n* is a positive integer or zero, *m* is an integer and *n* − |*m*| = even number, with |*m*| ≤ *n*; *r* is vector length from an origin point to a given target control point of surface $(x,y,z):\gamma =\sqrt{x^{2}+y^{2}+z^{2}}$, −1 < *x*, *y*, *z* < 1.

**Feature 5**

Consider

(11) $$\begin{array}{c} \varepsilon_{n_{1},n_{2}}=\frac{\max_{(u,v)V}\left|P\left(u,v\right)-P_{n_{1},n_{2}}\left(u,v\right)\right|}{\left|P\left(u,v\right)\right|}\times 100\%;\\ {\overset{-}{S}}_{n_{1},n_{2}}=1-\frac{\varepsilon -\varepsilon_{n_{1},n_{2}}}{\varepsilon_{n_{1}-1,n_{2}-1}-\varepsilon_{n_{1},n_{2}}}. \end{array}$$

Here *β*_*n*−1_(*t*) is the high-frequency surface obtained from the 1st order wavelet decomposition of *f*_*n*_(*t*).

## 4. Experiment and Computation

**Figure 1:**
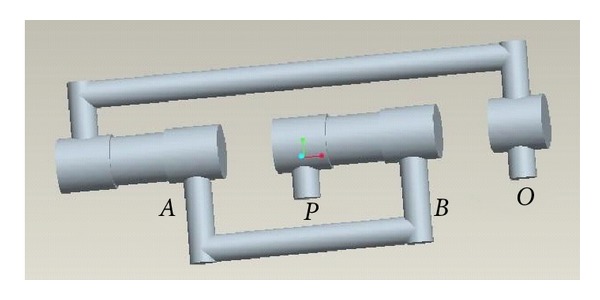
The constructed three dimensional model of target runner.

**Figure 2:**
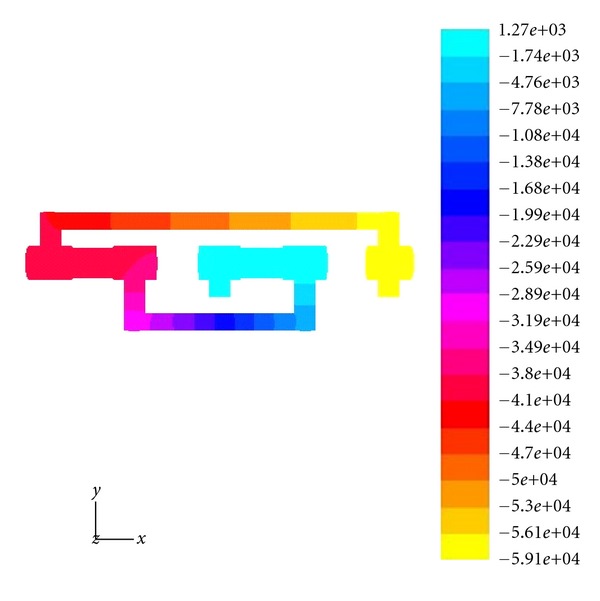
The distribution characteristics and change processes of turbulence kinetic energy in a high-pressure valve runner.

Figure 1 illustrates the constructed three-dimensional model of one given high-pressure reversing valve runner (Type No. D5-02-2B-AC-A01) by using PRO-E software, with its spatial structure gridded in fluent system.  Figure 2 denotes the distribution characteristics and change processes of turbulence kinetic energy in it, the values of kinetic energy illustrated by different color sections in the left column. First the required turbulence field is simulated with SNQ-1TX-140 microturbulence generator, and a produced PVC transparent valve runner is applied for clearly observing the detailed flow process. The specific experimental condition can be defined as follows: flow quantity is 10–20 Min/L, working pressure is higher than 20–30 MPa, flow velocity of flow field exit is faster than 10–30 cm/s, the spatial arrangement of valve runner is 150 mm × 150 mm × 30 mm, together with the experimental time duration being kept as long as 2–4 hours; all these condition parameters require precision adjustment in the interest of energy distribution modeling. 

**Figure 3:**
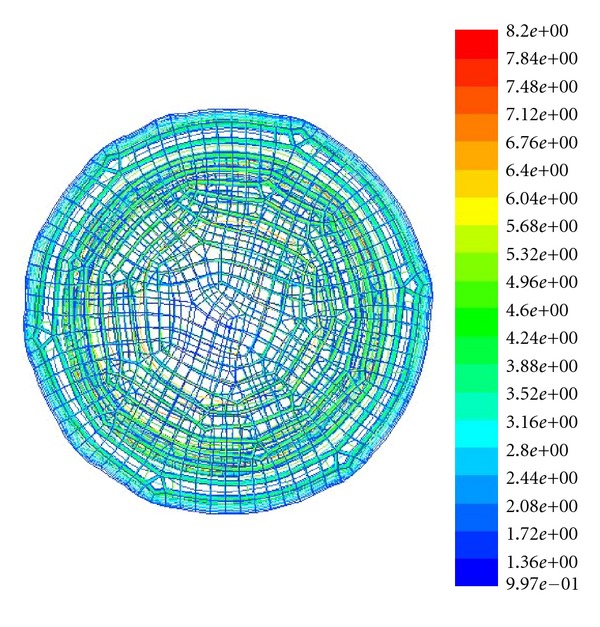
The gridded fluid section in the high-pressure reversing valve runner.

**Figure 4:**
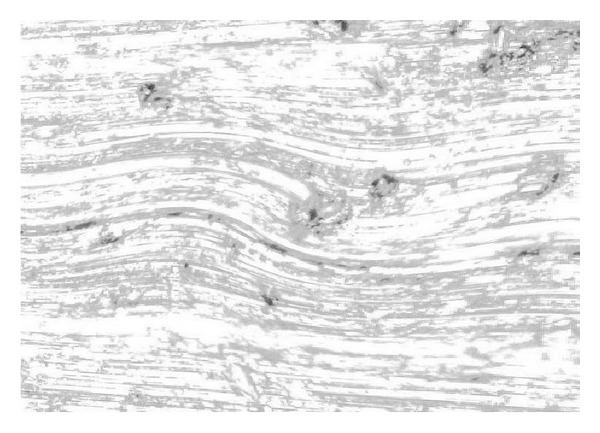
Turbulence in the runner of a high-pressure reversing valve.

As Reynolds number *Re* = *uh*/*v* is defined as 4700~4900, Figure 3 denotes the gridded fluid runner, and Figure 4 shows the turbulence imaging result. Through adopting finite volume method (FVM) in a staggered grid we implement a discretized data process on turbulence equation set. By positioning those monitor points that show key fluid parameters such as pressure *P*, dissipation rating *ε* at the center of grid boundary, and the monitor points of flow velocity *μ* on the grid boundary, we use a power function to parameterize the whole duration of data processing. 

The exit boundary pressures of turbulence field are supposed as identical to those of external environment, whose normal gradient value is normally determined as zero. For the purpose of describing the boundary influences emerging from turbulence field wall, we assume they are from a nonslip condition. Namely, the three-dimensional motion velocities at the objective positions of turbulence monitoring points *μ*_*i*_(*U*, *V*, *R*) are defined as Δ*S*_*pU*_ = −*A*_cell_Γ_wall_/*δ*_*p*_.

Here Δ*S*_*pU*_ denotes the corrected value of an original item, *A*_cell_ denotes the area of a boundary grid which parallels a flow field section, and Γ_wall_ denotes an effective exchanging coefficient of velocity components that normal to the runner wall [21].

Turbulence motion parameters such as flow velocities, pressures, and pressure intensities are calculated or measured at each grid monitory point in different boundary conditions; we compute turbulence kinetic energy *K* with one detailed representative calculating process in *k*-*ε* model, as described by (1)–(6).  Table 1 shows the computation process and result of comparison of turbulence parameter in different experimental condition by using *k*-*ε* model. Simultaneously an instantaneous flow tester from the state key laboratory for hydraulic control technology hosted at Guangzhou University is used for directly measuring turbulence velocities, motion directions, and determining kinetic energy on the above-mentioned section point, which facilitates the comparison between those measured results and computed ones in this table; through inspection of the deviation value between *K* and *K*_measured_ this newly proposed calculation method of turbulence kinetic energy can be verified [22].

Afterwards, in the desire of calibrating the distribution of those obtained kinetic energy values on the objective section, we mesh the whole section plane into 100 × 80 points in *x-*axis and*y*-axis, respectively, with consideration of practical conditions and precision requirements, as shown in Figure 3. 

**Figure 5:**
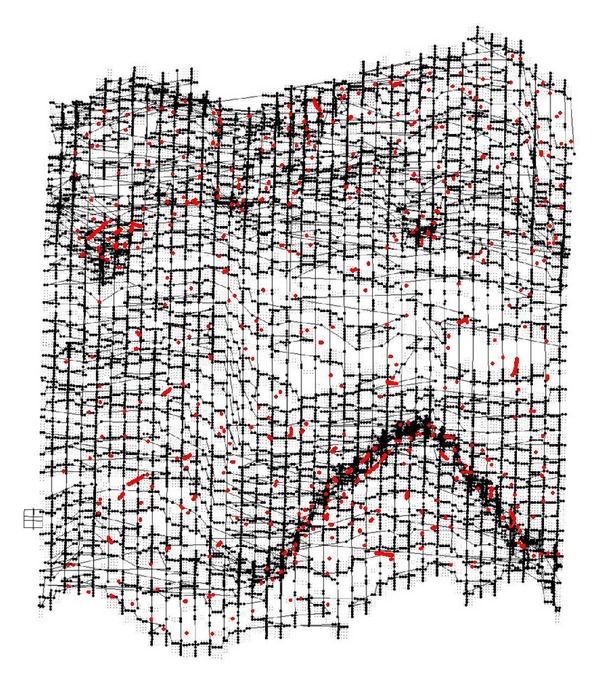
The point cloud of energy distribution section obtained by calculating kinetic energy values on each position point.

For describing the respective microfluid kinetic energy distribution on the objective runner, those inflection points representing their particular energy value with a symbolized significance are chosen as the surface control points, such as the coordinate points with boundary values, curvature change rules, corner values, salient values, discrete values, or stepping values as well [22]. As shown in Figure 5, when we regarded the turbulence kinetic energy value of one objective runner position point as a vertical coordinate of *z* axis which is perpendicular to its belonging section plane denoted by *x* and *y* ones, the three-dimensional visual point cloud of energy distribution can be obtained by calculating kinetic energy values, and some key position points with representative kinetic energy value (or control points in a geometrical sense) are highlighted in red, which helps to quantitatively evaluate the mutual fuzzy relation mechanism between energy distribution characteristics and surface fitting algorithms in a specific experimental condition. 

**Figure 6:**
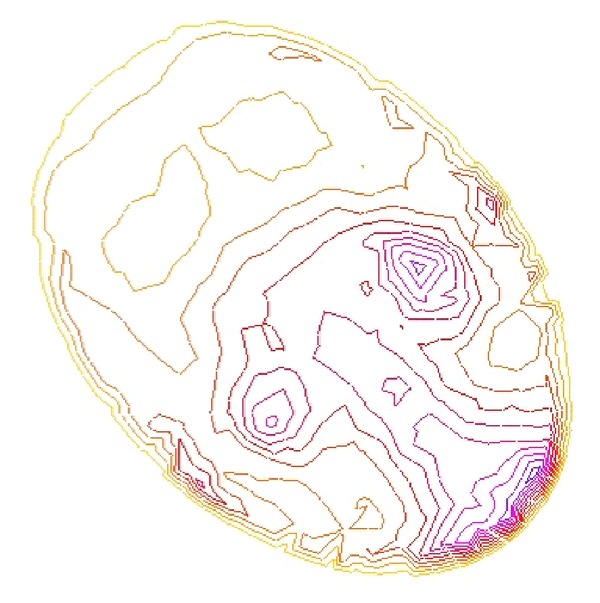
The kinetic energy value spatial distribution area in the objective runner.

**Figure 7:**
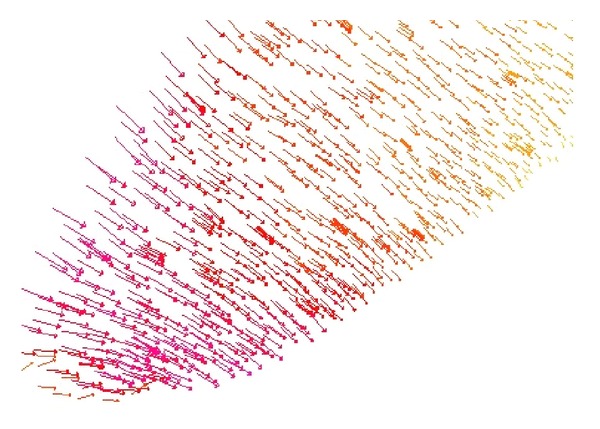
The dynamic transitional vectors of kinetic energy distribution.

**Figure 8:**
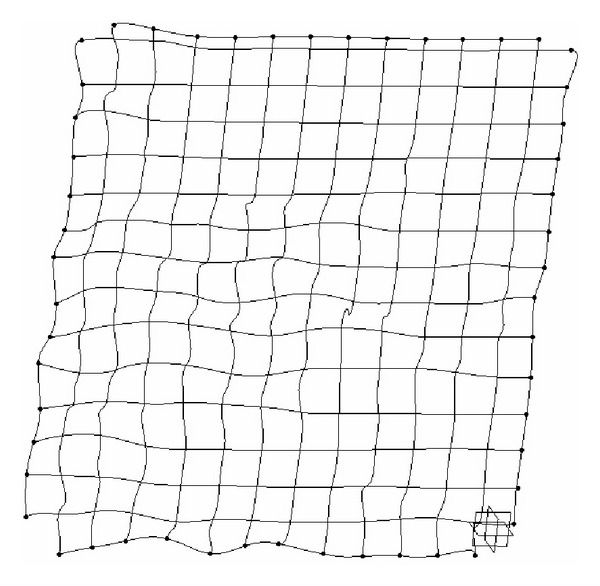
The skeleton frame of kinetic energy distribution surface.

**Figure 9:**
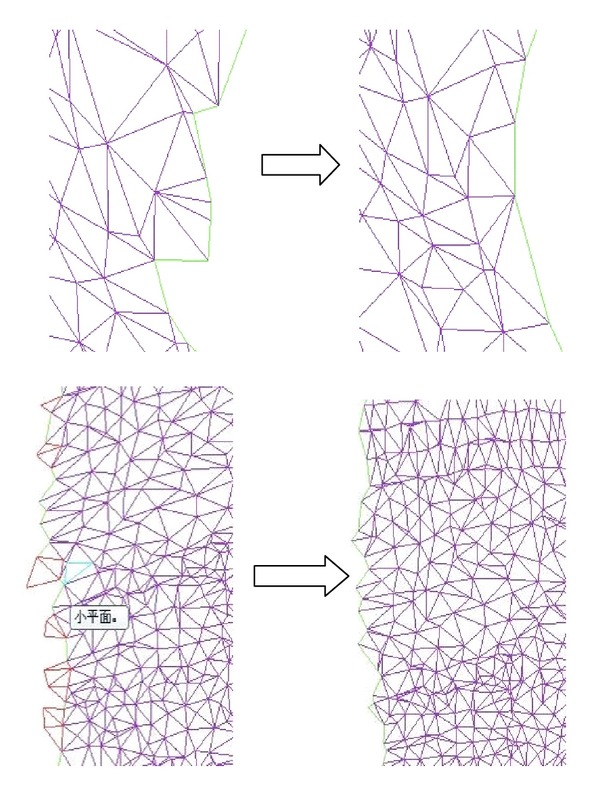
The smoothing operation of one specific spatial grid of target kinetic energy distribution surface model.

**Figure 10:**
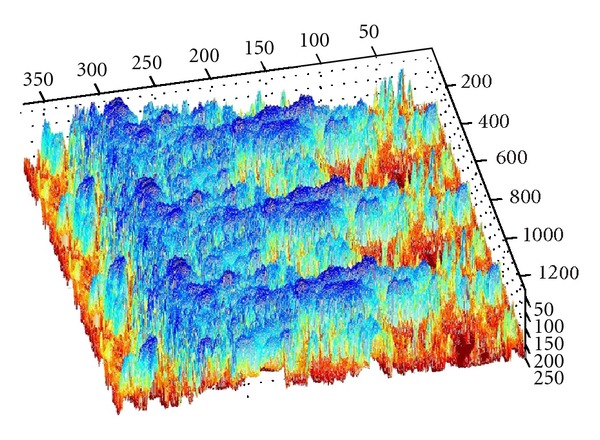
The constructed turbulence kinetic energy distribution surface of NURBS in high-pressure reversing valve runner.

**Figure 11:**
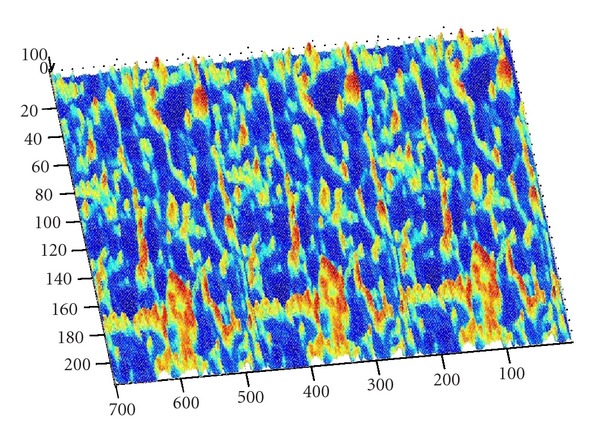
The constructed turbulence kinetic energy distribution surface of energy optimization modeling in high-pressure reversing valve runner.

**Figure 12:**
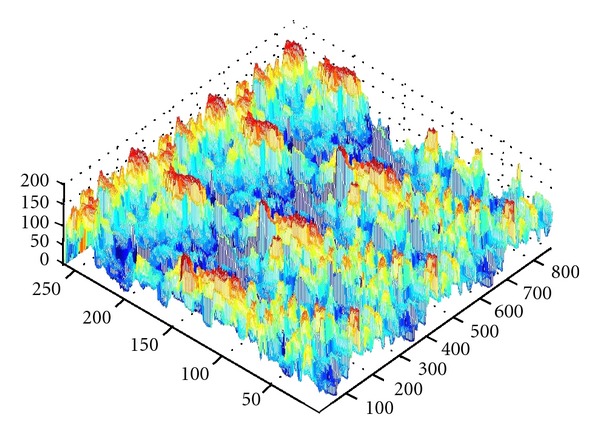
The constructed turbulence kinetic energy distribution B-spline surface of quasiuniform bicubic in high-pressure reversing valve runner.

**Figure 13:**
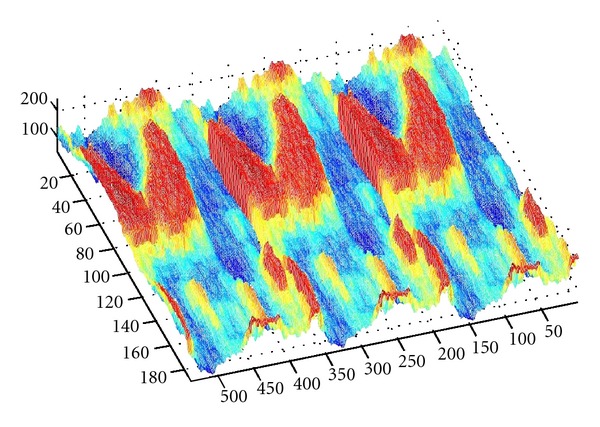
The constructed turbulence kinetic energy distribution surface of trigonometry Bernstein-Bezier in high-pressure reversing valve.

**Figure 14:**
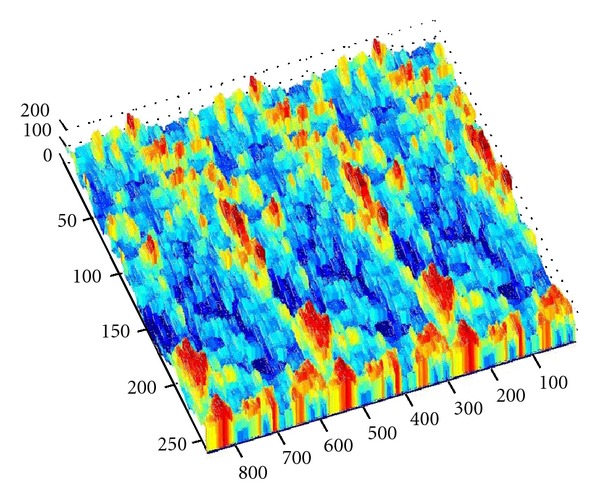
The constructed turbulence kinetic energy distribution surface of scattered data interpolation in high-pressure reversing valve runner.

As we denote a geometrical corner point as origin *O*, an absolute coordinate system *O*(*X*, *Y*, *Z*) is established. We import the kinetic energy values computed on the base of aforementioned algorithms; the energy values can be regarded as the *z*-axis coordinates.  Figure 6 shows the kinetic energy distribution in the objective runner obtained by experimental simulation, and Figure 7 shows the dynamic transitional vectors as well. Under the idea of guidance of this newly proposed concept, using Catia v5r19 we established the skeleton frame of kinetic energy distribution surface constructed by using key position points and the dynamic transitional vectors between them, as one skeleton patch as shown in Figure 8. Furthermore, in order to optimize the surface precision qualities, Figure 9 presents the smoothing operation of one spatial grid obtained from trigonometry Bernstein-Bezier method, which can be achieved on the base of wiping off the redundant small surface patches in boundary areas. Thus their respective energy distribution surface can be finally established; for example, the constructed turbulence kinetic energy distribution surface of NURBS on high-pressure reversing valve runner, with its energy values, is highlighted by different color areas in this figure, as Figure 10 shows, the same as energy optimization modeling surface (Figure 11), B-spline surface of quasiuniform bicubic (Figure 12), trigonometry Bernstein-Bezier surface (Figure 13), and scattered data interpolation surface (Figure 14); it is worth noting that the detailed surface fitting processes are abbreviated in the interest of an obvious limiting length and research focus of this paper. As the constructed surface is enclosed by the boundary control curves, we use *u* and *v* axes to denote the transverse and longitudinal orientations with their value scales being [0, 4000] and [0, 5000], respectively, (um). Simultaneously, as the result of the data value of the vertical *z* axis (*z* direction) has a completely different meaning from that of *u* and *v* axis, therefore the value range of kinetic energy is used to clearly indicate its stereo features and spatial surface details. All surface feature blocks are highlighted by pseudocolors in order to illustrate energy distribution details as well. 

In order to accurately quantify turbulence energy distribution with the help of these surfaces, by using (7)–(11) we determine the spatial mathematical features, with the mean values of these feature results being demonstrated in Tables 3, 4, 5, 6, and 7. On this basis a specific analysis and data evaluation can be successfully conducted as follows.

## 5. Three-Dimensional Fuzzy Performance Analysis and Evaluations

Table 2 defines the experimental parameters for different surface modeling methods. In this paper, we propose an improved three-dimensional fuzzy parameter system to establish a reliable influence evaluation mechanism as required. Different from those traditional ones, it does not require any previous information other than the three dimensional data to be disposed, but which needed by fuzzy ones [23]. Feature parameter sequence *f*_*i*_(*k*) can be determined as

(12) $$\begin{array}{c} {\text{feature}}_{i}\left(k\right)=\left(f_{i}\left(1\right),f_{i}\left(2\right),\ldots ,f_{i}\left(n\right)\right). \end{array}$$

Here *f*_*i*_(*k*) denotes the surface feature sequence parameters obtained from the aforementioned steps (objective sequence), *i* ∈ [1,2, 3,4, 5] denotes the number of surface features, and *k* denotes the sample surface blocks with their total number being *n*. On the other hand, the parameter sequence of modeling condition is illustrated as:

(13) $$\begin{array}{c} {\text{parameter}}_{i}=\left(tp_{i}\left(1\right),tp_{i}\left(2\right),\ldots ,tp_{i}\left(n\right)\right). \end{array}$$

Here *tp*_*i*_(*k*) denotes the condition feature sequence parameters (objective sequence), and *i* ∈ [1,2, 3,4, 5, 6, 7, 8, 9, 10, 11, 12] denotes the specific feature numbers [24–26]. 

The sequence of surface fitting methods is described as

(14) $$\begin{array}{c} {\text{method}}_{i}=\left(m_{i}\left(1\right),m_{i}\left(2\right),\ldots ,m_{i}\left(n\right)\right). \end{array}$$

Then we compute the fuzzy relation operator fuzzy_*i*_′(*k*, *i*) as follows, with which an integrated fuzzy relation matrix can be established:

(15) fuzzyi′(k,i) =mn∑k=jm∑r=in[featurei(k)−featurei(k)¯]∗𝒜∗[mi(k)−mi(k)¯]∑r=jm∑k=in[featurei(k)−featurei(k)¯]∑r=1m∑k=1n∗∑r=jm−j+1∑k=in−i+1[mi(k)−mi(k)¯]∗∑r=jm−j+1∑k=in−i+1𝒜,

where *𝒜* denotes [technical_parameter_*i*_(*k*)−$\bar{\text{technical}\_{\text{parameter}}_{i}(k)}]$. Here *k* = 1,2,…, *n*, *i* = 1,2,…, *m*, and $\bar{{\text{feature}}_{i}(k)}$, $\bar{\text{technical}\_{\text{parameter}}_{i}(k)}$, $\bar{m_{i}(k)}$ are the average function vectors of feature_*i*_(*k*) and technical_parameter_*i*_(*k*), *m*_*i*_(*k*), respectively. *m*_*i*_(*k*) denotes the surface fitting methods (reference sequence), and *i* ∈ [1,2, 3,4, 5] denotes the number of fitting methods.

The fuzzy relation coefficient *ƛ*_*i*_(*k*, *r*) between the approximate target and the practical surface can be calculated as follows:

(16) $$\begin{array}{c} {ƛ}_{i}\left(k,r\right)=\frac{\sum_{i-1}^{r}\left(\min_{i\in I}\;\min_{k}\left|\begin{array}{c} {{\text{fuzzy}}_{0}}^{*}\left(k,r\right)\\ -{{\text{fuzzy}}_{i}}^{*}\left(k,r\right) \end{array}\right|\right)+\beta \sum_{i=1}^{r}\left(\max_{i\in I}\;\max_{k}\left|\begin{array}{c} {{\text{fuzzy}}_{0}}^{*}\left(k,r\right)\\ -{{\text{fuzzy}}_{i}}^{*}\left(k,r\right) \end{array}\right|\right)}{\sum_{i=1}^{r}\left(\left|\begin{array}{c} {{\text{fuzzy}}_{0}}^{*}\left(k,r\right)\\ -{{\text{fuzzy}}_{i}}^{*}\left(k,r\right) \end{array}\right|\right)+\beta \sum_{i=1}^{r}\left(\max_{i\in I}\;\max_{k}\left|\begin{array}{c} {{\text{fuzzy}}_{0}}^{*}\left(k,r\right)\\ -{{\text{fuzzy}}_{i}}^{*}\left(k,r\right) \end{array}\right|\right)}. \end{array}$$

Here *β* is the distinguishing parameter set as 0.5~0.7. The details are shown in Tables 8–12, which illustrates the fuzzy performance mechanism between them in different experimental parameter conditions. 

With Table 8 it can be observed that NURBS fitting method exerts an obvious fuzzy relation influence on amendment quantity of external load and fairing error. And it is also highly impacted by the number of control points in *u* and *v* domain, number of boundary constraint vectors, and rank range of derivative coefficient matrix, and so forth. Energy optimization surface of turbulence kinetic energy distribution, as Table 9 shows, obviously keeps a rather close fuzzy relation with elasticity variance ratio and Zernike moment, and so forth. It is highly impacted by the number of boundary constrain vectors, order of normal vectors, and kinetic energy coefficient of external loading. Quasiuniform bicubic B-spline surface of turbulence kinetic energy (Table 10), markedly keeps close fuzzy relation with energy dispersive-ratio or faring error in the proposed experimental parameter conditions. It can be affected by the number of boundary constrain vectors, order of knot vector, and number of boundary constrain vectors, and so forth. The Bernstein-Bezier surface used for fitting turbulence kinetic energy distribution, as Table 11 demonstrates, obviously exerts a fuzzy influence on elasticity variance ratio and amendment quantity of external load. Scattered data interpolation used for turbulence kinetic energy distribution models, as shown by Table 12, keeps a close fuzzy relation with energy-dispersive ratio and amendment quantity.

Table 13 shows the performance comparisons of these proposed surface fitting algorithms in the whole experimental process. With its detailed demonstration we conclude that trigonometry bicubic B-spline fitting method has a wide application in a characteristic surface fitting condition that puts greater emphasis on the accuracy rate and shape precision of energy distribution; with the similar evaluation approach we observe that B-spline surface of quasiuniform bicubic will be more suitable for constructing a simpler approximate fluid energy distribution model; trigonometry Bernstein-Bezier ensures the high fitting accuracy and control precision of knot vectors and surface models; finally we achieve a good experimental process in computation time, computation storage, and approximate error when using scattered data interpolation. After data comparison and detailed analysis we found that energy optimization surface modeling gets an optimum integrated performance capability in practice and therefore becomes our preferred choice.

The following suggestions are proposed for developing turbulence energy distribution modeling. (1) In the interest of obtaining accurate coordinate information it is suggested that the distribution intensity when selecting position points with representative kinetic energy values (or being called the control points in geometrical modeling sense) should be in a medium-low level from 20 points per mm^2^ to 40 points per mm^2^, the function order of knot vectors or boundary constrain vectors be kept in a relatively low state from three to four, and the signal probing frequency of coordinate acquirement be lower than 300 times/minute, which will strongly support for a high fidelity reflection of energy distribution characteristics in a detailed pattern. (2) Fluid kinetic energy distribution properties keep a close fuzzy relation with their inherent surface fitting qualities and practical measuring parameters, with the analysis details being clearly shown in the previously mentioned paragraphs. (3) It is proposed that the three-dimensional fuzzy performance mechanism possessed by these typical energy distribution surface fitting methods be quantized by an explicit mathematical expression through detailed experimental evaluations. (4) We can make a final assessment on the specific applications of typical surface fitting algorithms when dealing with turbulence kinetic energy distribution problems: NURBS can be widely used in a preliminary characteristic classification of turbulence kinetic energy distribution; energy optimization can be used to promulgate or improve the distribute rationality of modeling information and the strain intensity of kinetic energy signal illustration, which becomes our top-preferred selection item in this experiment; quasiuniform bicubic B-spline can be conveniently used for optimizing the energy details of those newly constructed turbulence energy distribution; Bernstein-Bezier has been frequently used in the shape optimization of energy distribution microsurface when an external dynamic energy loading is exerted; we can also use scattered data interpolation to produce a more robust surface model of energy distribution when facing with some noise point coordinates (position points with useless or misleading kinetic energy information or things like that) and truncation errors during data acquisition as well; all these suggestions provide a valuable reference and improve evaluation practices for solving all similar problems in the future.

## 6. Conclusions

This paper sought to investigate the fuzzy performance mechanism exerted by surface fitting algorithms on the constructed turbulence kinetic energy distribution models in different experimental parameter conditions. With a newly proposed three dimensional fuzzy relation evaluation method, we verified a series of quantified turbulence energy distribution surface features to analyze the complicated fuzzy relation mechanism between them. 

This investigation has the following theoretical superiorities over other traditional researches For the traditional methods simply focused on establishing a turbulence energy distribution model without any further considerations about its spatial distribution surface, the surface fitting algorithm, and its consequent impact on turbulence energy modeling results, we are concerned with the mutual-performance mechanism and uncertainty principle from miscellaneous data analysis; different from other traditional ones in concluding turbulence energy distribution properties on one given high-pressure fluid field from macroscale dimensional analysis, we proposed a new three dimensional fuzzy performance mechanism of surface fitting and realized its resulting quantization by discussing the microturbulence characteristic details in an experimental condition; considering the absence of fuzzy relation calibration between turbulence energy distribution and surface fitting in a traditional research, we investigated their internal mutual-performance mechanism and then assessed the respective fuzzy influence factors and inherent mathematical principles as respected. 

The following major contributions are included in our work. As the traditional method has not touched upon turbulence kinetic energy distribution surface on one reversing valve's high-pressure runner, we proposed several new mathematical features to accurately show the objective surface and quantitatively evaluated their inherent features in geometrical domain; through using surface fitting for modeling turbulence kinetic energy distribution in a geometrical domain, we analyzed and quantified the fuzzy influences of surface fitting on the constructed energy distribution surface models in different experimental conditions, with their inherent change rules also being clearly indicated; we proposed an improved three dimensional fuzzy relation evaluation system to establish reliable performance mechanism which does not require any previous information other than the experimental data to be disposed, and thereafter an in-depth discussion about fuzzy performance has been made. And finally, several original suggestions concerning the specific surface fitting processes and their fuzzy performance in geometrical surface domain and turbulence energy distribution sense have been presented as well.

All the above-mentioned processes could be successive stages of computation and analysis, with the second operating on the output of the first. It solves the difficulties and dilemma in quantitatively assessing an optimum fuzzy performance evaluation method or surface fitting algorithm when researching turbulence energy distribution characteristics on one high-pressure reversing valve runner. For the experimental process has a deliberate theoretical foundation, thus the mathematical analysis process can be founded and simplified, and this research also provides a new idea for following turbulence characteristic quantitative evaluation.

## Figures and Tables

**Table 1:** Computation process and result comparison of turbulence parameter in different experimental condition by using *k*-*ε* model.

*μ* (cm/s)	*ε* (cm^2^/s^3^)	*K*_th_ (cm^2^/s^2^)	*K* (cm^2^/s^2^)	*K*_measured_ (cm^2^/s^2^)	Deviation value (cm^2^/s^2^)
5	2.75 × 10^−3^	3418.23	4351.19	6278.24	1927.05
10	7.43 × 10^−2^	4759.12	6183.17	7187.69	1004.52
15	8.75 × 10^−3^	6418.23	6351.19	6278.24	−72.95
20	9.33 × 10^−2^	7359.12	8183.17	8487.69	304.52
25	0.244	10795.16	10698.33	11627.49	929.16
30	0.343	12697.26	11876.24	11394.06	−482.18
35	0.544	18795.16	17698.33	17627.49	70.84
40	0.943	37697.26	40876.24	41394.06	517.82

**Table 2:** External experimental parameters for different surface modeling methods.

Number of control points (*u*)	400	600	800	1000	1200	1400	1600	1800
Number of control points (*v*)	400	600	800	1000	1200	1400	1600	1800
Order of spline surface	1		2		3		4	
Order of knot vector	1		2		3		4	
Order of normal vectors	1		2		3		4	
Order of derivative vectors	1		2		3		4	
Number of constraint vectors	0	2	4	6	8	10	12	14
Order of constraint vectors	1		2		3		4	
Order of continuous level	1		2		3		4	
Kinetic energy coefficient	1.0	1.5	2.0	2.5	3.0	3.5	4.0	4.5
Rank of derivative matrix	10	20	30	40	50	60	70	80
Approximate error range	0.4	0.5	0.6	0.7	0.8	0.9	0.95	1.0

**Table 3:** Energy distribution surface mathematical features in NURBS surface.

No.	Feature 1	Feature 2	Feature 3	Feature 4	Feature 5
a	253.4	1250.3	896.4	2254.1	654.2
b	452.1	1147.6	887.6	2269.5	669.5
c	336.5	1584.6	992.4	2547.1	698.5
d	239.8	1325.8	865.4	2365.4	447.5
e	339.5	2014.6	857.4	3026.9	853.2
f	458.6	1854.3	887.4	3365.1	602.5
g	447.2	2231.0	836.5	3022.8	598.7
h	369.5	2014.8	884.9	3369.4	558.4
i	147.8	1578.9	759.5	3157.4	569.5
j	664.6	1148.9	771.4	4012.6	559.4
k	669.8	1574.8	725.6	3995.5	554.7
l	558.7	1369.5	771.2	3624.5	563.9

**Table 4:** Energy distribution surface features in energy optimization modeling surface.

No.	Feature 1	Feature 2	Feature 3	Feature 4	Feature 5
a	336.5	1458.7	889.7	2014.5	447.8
b	395.8	1169.5	895.4	2236.5	456.9
c	402.5	1785.5	887.5	2541.8	552.4
d	448.6	1126.6	886.9	2214.7	568.4
e	451.2	1255.4	892.6	2365.9	556.3
f	395.8	2014.4	902.5	2548.5	529.6
g	336.5	2230.1	911.4	2547.8	547.8
h	365.7	1254.6	875.6	2364.5	551.2
i	602.5	1138.6	884.5	3014.5	536.9
j	605.4	1204.5	902.6	2893.2	547.8
k	663.1	1169.5	923.5	2965.4	548.9
l	625.4	1247.5	933.6	2268.2	557.4

**Table 5:** Energy distribution surface features in B-spline quasiuniform bicubic.

	Feature 1	Feature 2	Feature 3	Feature 4	Feature 5
a	625.4	1478.6	1024.5	2014.7	698.5
b	557.8	1159.6	1152.3	2233.6	815.2
c	512.6	1203.6	1045.6	2014.5	478.6
d	654.3	1475.2	1102.3	2159.8	556.9
e	478.5	1149.8	1069.8	2306.5	547.8
f	551.2	1523.6	1024.5	2147.8	558.6
g	526.3	1402.5	1147.6	2236.5	526.3
h	554.7	1163.2	1036.5	2105.6	547.2
i	625.4	1299.8	1029.6	2452.3	551.2
j	663.2	1475.6	1302.5	2144.5	529.8
k	602.5	1254.6	1336.5	2036.5	547.8
l	663.1	1178.9	1025.4	2045.6	553.6

**Table 6:** Energy distribution surface features in trigonometry Bernstein-Bezier surface.

	Feature 1	Feature 2	Feature 3	Feature 4	Feature 5
a	712.5	1214.5	1025.6	2147.5	558.6
b	658.6	1147.8	998.5	2236.5	893.6
c	669.5	1326.5	952.6	2014.6	602.5
d	625.4	1145.6	869.5	2514.6	621.5
e	615.4	1026.3	884.5	2014.5	548.6
f	639.5	1058.4	872.6	2231.5	554.7
g	702.5	1024.5	893.6	1987.5	523.6
h	742.3	1136.5	902.6	2036.5	547.8
i	605.4	1024.5	993.6	2245.6	523.2
j	663.5	1203.6	924.5	2011.4	514.8
k	685.2	1147.5	923.6	2036.5	556.3
l	635.4	1258.6	914.5	1877.5	529.6

**Table 7:** Energy distribution surface features in scattered data interpolation surface.

	Feature 1	Feature 2	Feature 3	Feature 4	Feature 5
a	478.9	998.5	1125.6	1995.6	635.8
b	448.5	895.6	1136.2	1897.5	665.4
c	524.6	885.4	1025.4	1785.6	639.5
d	485.6	875.6	995.6	1887.9	624.7
e	485.2	932.5	984.5	1987.6	702.5
f	402.6	902.6	975.2	1988.6	711.6
g	446.2	887.5	996.5	2015.6	711.3
h	475.8	893.6	924.5	2114.5	589.6
i	425.6	845.2	968.5	1899.6	588.9
j	485.2	869.3	889.6	1955.6	602.3
k	441.5	887.5	1021.3	2036.5	633.1
l	442.6	879.6	1125.4	1988.6	654.9

**Table 8:** Fuzzy relation degrees between experimental condition parameters and kinetic energy distribution surface features in the form of NURBS surface.

Condition	Feature 1	Feature 2	Feature 3	Feature 4	Feature 5
Number of control points (*u*)	0.8956	0.9254	0.4478	0.5849	0.6589
Number of control points (*v*)	0.6254	0.9125	0.5963	0.8965	0.5542
Order of spline surface	0.2546	0.9632	0.9254	0.8475	0.6235
Order of knot vector	0.3365	0.8654	0.9125	0.4521	0.8452
Order of normal vectors	0.2547	0.7748	0.6589	0.9214	0.5742
Order of derivative vectors	0.4156	0.7639	0.4785	0.8523	0.6214
Number of constrain vectors	0.6215	0.5482	0.9921	0.6214	0.8523
Order of constrain vectors	0.2558	0.1254	0.8848	0.6144	0.9214
Order of continuous level	0.5478	0.3654	0.6215	0.5248	0.5426
Kinetic energy coefficient	0.3654	0.8457	0.5144	0.6395	0.3654
Rank of derivative matrix	0.5523	0.3369	0.4478	0.6235	0.5846
Approximate error range	0.6245	0.8452	0.9542	0.5145	0.6695

**Table 9:** Fuzzy relation degrees between experimental condition parameters and kinetic energy distribution surface features in the form of energy optimization modeling surface.

Condition	Feature 1	Feature 2	Feature 3	Feature 4	Feature 5
Number of control points (*u*)	0.1542	0.5547	0.6354	0.2254	0.3654
Number of control points (*v*)	0.6635	0.6235	0.5524	0.1687	0.5547
Order of spline surface	0.2587	0.5842	0.4852	0.6245	0.6325
Order of knot vector	0.9856	0.3369	0.2653	0.6952	0.9236
Order of normal vectors	0.8854	0.3158	0.4875	0.9025	0.2254
Order of derivative vectors	0.7536	0.4758	0.2654	0.9125	0.5784
Number of constraint vectors	0.6658	0.6125	0.5524	0.2458	0.2654
Order of constraint vectors	0.6235	0.4857	0.6932	0.6214	0.2036
Order of continuous level	0.4859	0.6235	0.9245	0.6254	0.5147
Kinetic energy coefficient	0.3698	0.5547	0.8954	0.3652	0.2658
Rank of derivative matrix	0.9325	0.2584	0.8214	0.3924	0.4852
Approximate error range	0.9254	0.2415	0.6352	0.9226	0.6214

**Table 10:** Fuzzy relation degrees between experimental condition parameters and kinetic energy distribution surface features in B-spline surface of quasiuniform bicubic.

Condition	Feature 1	Feature 2	Feature 3	Feature 4	Feature 5
Number of control points (*u*)	0.5524	0.3958	0.2658	0.5578	0.5547
Number of control points (*v*)	0.1025	0.6589	0.6354	0.6247	0.6524
Order of spline surface	0.2514	0.9254	0.8547	0.5478	0.6635
Order of knot vector	0.4152	0.9254	0.2458	0.6692	0.5874
Order of normal vectors	0.5598	0.6354	0.3654	0.5896	0.4025
Order of derivative vectors	0.9025	0.4258	0.9221	0.6254	0.2214
Number of constraint vectors	0.9254	0.4475	0.9223	0.6698	0.6354
Order of constraint vectors	0.9125	0.5263	0.6245	0.8547	0.2954
Order of continuous level	0.2635	0.3958	0.5587	0.9365	0.6245
Kinetic energy coefficient	0.2875	0.6322	0.8475	0.2584	0.2258
Rank of derivative matrix	0.6395	0.1475	0.6354	0.6635	0.1587
Approximate error range	0.9254	0.5874	0.6698	0.1547	0.6325

**Table 11:** Fuzzy relation degrees between experimental condition parameters and kinetic energy distribution surface features trigonometry Bernstein-Bezier surface.

Condition	Feature 1	Feature 2	Feature 3	Feature 4	Feature 5
Number of control points (*u*)	0.8831	0.5521	0.6311	0.5538	0.9831
Number of control points (*v*)	0.8836	0.7732	0.7713	0.8831	0.9042
Order of spline surface	0.6637	0.5572	0.6637	0.6618	0.7748
Order of knot vector	0.6648	0.6618	0.9057	0.5173	0.1623
Order of normal vectors	0.6627	0.1649	0.1724	0.8946	0.0641
Order of derivative vectors	0.3326	0.4621	0.5731	0.6681	0.8862
Number of constraint vectors	0.8845	0.1713	0.5538	0.7852	0.5824
Order of constraint vectors	0.6411	0.4813	0.5294	0.7524	0.7711
Order of continuous level	0.6411	0.6582	0.9057	0.7742	0.6630
Kinetic energy coefficient	0.7842	0.8952	0.9083	0.6638	0.6491
Rank of derivative matrix	0.6381	0.6749	0.7482	0.8849	0.7381
Approximate error range	0.6481	0.7749	0.8940	0.7759	0.7391

**Table 12:** Fuzzy relation degrees between experimental condition parameters and kinetic energy distribution surface features in scattered data interpolation surface.

Condition	Feature 1	Feature 2	Feature 3	Feature 4	Feature 5
Number of control points (*u*)	0.2145	0.5874	0.5596	0.5587	0.5526
Number of control points (*v*)	0.6332	0.8214	0.8254	0.7154	0.4475
Order of spline surface	0.6254	0.9025	0.6325	0.4896	0.8215
Order of knot vector	0.2014	0.6684	0.7152	0.8854	0.6635
Order of normal vectors	0.6005	0.7025	0.9025	0.8256	0.5547
Order of derivative vectors	0.3958	0.1547	0.8869	0.7742	0.5596
Number of constraint vectors	0.5874	0.3654	0.1452	0.9025	0.1475
Order of constraint vectors	0.3369	0.5586	0.6054	0.4856	0.6524
Order of continuous level	0.9021	0.4852	0.6625	0.5514	0.5589
Kinetic energy coefficient	0.3654	0.6635	0.7012	0.6325	0.9325
Rank of derivative matrix	0.8878	0.9201	0.5584	0.4475	0.6635
Approximate error range	0.9356	0.5547	0.9245	0.5589	0.5548

**Table 13:** Performance comparison of different surface fitting algorithms during the modeling processes of turbulence kinetic energy distribution.

Algorithms	Surface distortion rate	Computation time	Approximate error	Computation storage
NURBS surface	3.254%	44.5 s	6.55%	4475.6 kb
Energy optimization modelling	6.225%	36.5 s	4.15%	33025.4 kb
B-spline quasi-uniform bicubic	4.115%	39.5 s	6.99%	11475.2 kb
Trigonometry Bernstein-Bezier	5.114%	62.5 s	3.58%	8896.5 kb
Scattered data interpolation	6.226%	44.5 s	4.77%	9924.5 kb
